# Blood–CSF MiRNA Signatures in Alzheimer’s Disease Reveal Novel Biomarker Candidates: A Systematic Review

**DOI:** 10.3390/ijms27219506

**Published:** 2026-10-24

**Authors:** Asmae Benchohra, Cristina Juárez-Alía, Gemma Serrano, Tomás Segura, Juan R. Peinado, Yoana Rabanal-Ruiz

**Affiliations:** 1 Molecular Studies Applied to Health Sciences Group (EMAS), Department of Medical Sciences, Faculty of Medicine of Ciudad Real, Institute of Biomedicine (IB-UCLM), University of Castilla-La Mancha, Paseo de Moledores s/n, 13071 Ciudad Real, Spain; asmae.benchohra@uclm.es (A.B.); cjuarezalia@gmail.com (C.J.-A.); 2 Multidisciplinary Group in Psychiatry and Translational Neuroscience, Health Research Institute of Castilla-La Mancha (IDISCAM), 45071 Toledo, Spain; 3 Department of Neurology, General University Hospital of Albacete, 02008 Albacete, Spain; gemmas@sescam.jccm.es (G.S.); tomas.segura@uclm.es (T.S.); 4 Research Group on Cerebrovascular, Neurodegenerative and Neuro-Oncological Diseases, Health Research Institute of Castilla-La Mancha (IDISCAM), 45071 Toledo, Spain

**Keywords:** Alzheimer’s, biomarkers, CSF, blood, miRNAs

## Abstract

Alzheimer’s disease (AD) is a progressive neurodegenerative disorder characterized by cognitive decline. MicroRNAs (miRNAs) have emerged as promising regulators of AD-related pathways and potential minimally invasive biomarkers. However, existing meta-analyses have yielded inconsistent results, and whether miRNA signatures are conserved across biological fluids remains unclear. The present work aimed to systematically review studies performing miRNA discovery or multi-candidate profiling in blood and CSF during the last 15 years, in order to identify miRNAs showing significant and consistent differential expression across independent studies. By systematically comparing the direction and consistency of miRNA dysregulation across independent studies and biological sources, we identified 80 miRNAs consistently dysregulated in both blood and CSF, revealing a reproducible cross-fluid signature of AD-associated molecular alterations. Notably, miR-451a and miR-16-5p emerged among the most consistently dysregulated miRNAs across fluids. In contrast, several miRNAs displayed divergent or compartment-specific patterns, highlighting that blood and CSF may capture distinct aspects of AD-related molecular changes. Among these, miR-484, miR-3200-3p and miR-598 were identified as novel CSF candidates, while miR-20a-5p, miR-363-3p and miR-5006-3p represented novel blood-specific candidates. Furthermore, a substantial proportion of detected miRNAs was associated with extracellular vesicle/exosomal fractions, supporting their potential stability and biological relevance. Overall, our findings demonstrate that AD-associated miRNA dysregulation comprises both conserved and biofluid-specific signatures. From a clinical perspective, the current evidence primarily supports further evaluation of miRNAs as diagnostic/discriminative biomarkers rather than validated tools for early detection, disease monitoring, or prognosis. Their clinical translation remains limited by biofluid and methodological heterogeneity and requires harmonized cross-cohort and longitudinal validation.

## 1. Introduction

Alzheimer’s disease (AD) represents the most prevalent type of dementia and is characterized as a chronic neurodegenerative disorder affecting the brain [1]. In addition to minimally invasive diagnostic approaches, current strategies for the detection and monitoring of AD include the development of biomarkers through metabolomic and proteomic methodologies [2,3].

The discovery of biomarkers in biological fluids of AD and other neurodegenerative disorders has proven to be one of the most effective approaches to identify prognostic targets and to monitor disease progression [4,5]. Accordingly, several proteins have already been identified as potential biomarkers for AD [6,7].

Notwithstanding this, it has been extensively described that epigenetic factors play an important role in AD pathogenesis. Among them, the regulatory effects of microRNAs (miRNAs), small, non-coding RNA molecules that regulate gene expression at the posttranscriptional level, have been linked to underlying pathogenic pathways of AD, and various approaches have also been undertaken to identify miRNAs in blood and cerebrospinal fluid (CSF), which may not only serve as reliable biomarkers for AD diagnosis but also provide insights into the dysregulation of AD-associated protein expression [8].

miRNAs have several characteristics that make them attractive biomarker candidates in AD. They are detectable and relatively stable in blood and CSF, particularly when associated with extracellular vesicles or RNA-binding complexes [9], and their regulatory functions may reflect alterations in AD-related pathways, including amyloid processing, tau-related mechanisms, neuroinflammation, and synaptic dysfunction [8]. The evidence included in this review has primarily assessed their ability to discriminate patients with AD from cognitively healthy individuals, supporting their potential mainly as diagnostic/discriminative biomarkers.

miRNA-based biomarkers should therefore be considered complementary rather than alternative to established biomarkers. While Aβ42/Aβ40 and phosphorylated tau reflect core AD pathology and total tau and NfL provide information on neuronal injury or neurodegeneration [10,11], miRNAs may provide an additional regulatory and pathway-related molecular layer. Their added clinical value, however, still requires validation in standardized assays, independent cohorts, and longitudinal studies.

Despite substantial interest in this field, the results of miRNA studies that have examined plasma or CSF miRNA concentrations in AD have been inconsistent. Therefore, it remains unclear which specific miRNAs are most strongly associated with the disease, particularly in terms of reproducible differential expression between AD patients and healthy individuals. In addition, the body of available evidence is difficult to interpret due to significant methodological variability among studies, such as limited sample sizes and heterogeneous analytical approaches. These limitations not only hinder cross-study comparisons but also highlight the need to focus on biomarkers that can be reliably detected and validated across both blood and CSF.

The present work aimed to conduct a systematic review of all the studies that perform miRNA discovery in the literature during the last 15 years in both biological fluids in order to identify significant and consistent differential expression across publications. Most studies have focused on blood miRNAs, which are readily accessible, minimally invasive, and suitable for repeated sampling and large-scale clinical application, although circulating miRNA profiles may also reflect peripheral tissues and systemic processes. In contrast, CSF is more invasive to obtain but, given its close interaction with the central nervous system (CNS) and its established use for Aβ and tau biomarker assessment, may more directly reflect brain-related molecular alterations and potentially provide greater CNS specificity. However, it remains unclear to what extent AD-associated miRNA signatures are conserved across these two compartments or are biofluid-specific. In this context, identifying miRNAs reproducibly detected in both blood and CSF could improve biomarker reliability by combining biological relevance with clinical accessibility, whereas divergent patterns may provide complementary information on compartment-specific disease processes [12]. Our work highlights 80 unique miRNAs that consistently appear to be over- or under-represented in AD across independent studies. This integrative model not only reveals putative non-invasive biomarkers in several compartments but also sheds light on the miRNA-protein-disease network that could facilitate an improved understanding of the molecular processes associated with AD. Ultimately, these findings may positively influence the development of miRNA-based diagnostic tests and underscore the utility of cross-compartmental validation in biomarker discovery.

## 2. Methods

### 2.1. Eligibility Criteria

The review question was formulated according to the PICOS framework. The population comprised individuals with AD; the index of interest was miRNA expression in blood-derived samples or CSF; the comparator consisted of age-matched cognitively healthy controls; the primary outcomes were differential miRNA expression, direction of dysregulation, biofluid distribution, and reproducibility across independent studies; and eligible study designs included human observational studies using discovery or multi-candidate miRNA profiling approaches.

Eligible studies were limited to those published within the last 15 years and comprised studies examining differential miRNA expression in blood or CSF samples obtained from individuals with AD compared with healthy controls. Studies that evaluated blood-cell or extracellular vesicle-associated miRNAs derived from these biological fluids were also considered. Studies on animals, cell culture, and tissues, or those that offered qualitative results rather than differential miRNAs expression in AD patients’ blood or CSF, were disregarded. Because the primary objective of this systematic review was to identify miRNAs emerging from biomarker-discovery or multi-candidate profiling approaches, rather than to evaluate evidence for preselected individual miRNAs, only studies assessing at least three miRNAs were considered eligible. Studies exclusively investigating one or two candidate miRNAs were therefore excluded to reduce candidate-selection bias, as such studies are generally designed to test miRNAs already implicated in AD or selected on the basis of prior biological or clinical evidence, investigator-driven hypotheses, or laboratory-specific research priorities.

We considered all studies using any robust quantitative analytical method capable of identifying miRNAs associated with or discriminating between AD patients and healthy controls, including parametric and non-parametric statistical tests, regression models, and machine learning approaches.

For the syntheses, studies were grouped according to biological source (blood, CSF, or both). miRNAs were subsequently classified according to whether they were identified in both biological fluids or only in one source, and according to their reported direction of change in AD.

### 2.2. Information Sources and Search Strategy

A systematic literature search was performed in accordance with PRISMA guidelines to identify AD-associated microRNAs detectable in blood and CSF. PubMed was used as the information source. No language restrictions were applied. The literature search was conducted in two stages: an initial search was performed in February 2025, followed by an updated search in March 2026, using the following terms and excluding reviews and systematic reviews through the search syntax:

(“Alzheimer disease” OR “AD”) AND (“Human*” OR “Patient*”) AND (“serum” OR “plasma” OR “blood” OR “CSF”) AND (“miRNA*” OR “microRNA*” OR “micro-RNA*”) NOT (Review [Publication Type]) NOT (Systematic Review [Publication Type]).

### 2.3. Study Selection Process

The search results were imported into Rayyan AI, a web-based tool that facilitates screening and selection of studies in systematic reviews (https://www.rayyan.ai/, accessed 26 February 2026). Duplicates were removed, and each record was screened independently by two reviewers against the defined eligibility criteria. Studies with concordant inclusion decisions were retained. Discrepancies between the two reviewers were resolved through discussion involving additional reviewers, with more than two reviewers participating in the final decision when required, until consensus was reached. During the initial screening of the search results, each study was annotated on Rayyan AI, with labels and notes based on the defined criteria. These annotations later became a basis for a secondary screening in which studies were systematically classified as included or excluded.

### 2.4. Data Extraction

Main text and associated Supplementary Material of the included studies were examined to extract the predefined information on dysregulated miRNAs in CSF and blood of AD samples, depending on where the relevant results were reported. Data were extracted independently by two reviewers using the predefined extraction criteria. Discrepancies were resolved by discussion and consensus, with consultation of a third member of the review team when necessary. The data were compiled in a structured main dataset and in Supplementary Table S1A, where the section or source from which the miRNA findings were retrieved was recorded for each study. Study-specific selection decisions and clarifying notes were also recorded whenever additional criteria were required for data extraction.

Our main dataset was constructed by listing the miRNAs reported in each study as discriminating AD from controls, together with their direction of change in AD samples (“Increased” or “Decreased”) and their sample origin (“Blood” or “CSF”). These variables constituted the main outcome data sought for the review. For each study, we also recorded the study design (discovery or targeted analysis, and the type of validation, when performed), the specific sample type from which the miRNAs were obtained (fluid, cells, or extracellular vesicles), the technology used for miRNA detection (qRT-PCR, microarray, or small RNA-seq), cohort size and age, the neuropsychological criteria used to define the diagnostic groups (e.g., National Institute on Aging-Alzheimer’s Association guidelines), whether APOE genotype was considered, the statistical and computational methods used to identify miRNAs discriminating AD from controls, and the section from which the reported miRNAs were retrieved (Supplementary Table S1A).

Importantly, we prioritized high-confidence findings over preliminary signals. Accordingly, in studies including both discovery and validation phases, only miRNAs confirmed in the validation stage were extracted. However, discovery-phase results were considered when the validation stage did not confirm any miRNAs [13,14,15], was performed in a different sample type [16], or consisted solely of a comparison with findings from another study included in the review [17]. Additionally, when both uncorrected and corrected *p*-values were reported, we preferentially selected miRNAs meeting the corrected significance threshold [14]. Likewise, although some studies reported significance thresholds above 0.05, we retained only miRNAs with *p*-values < 0.05 whenever possible [18,19]. Clarifying notes were added to Supplementary Table S1A whenever additional study-specific selection criteria were applied.

When age was not reported as mean ± SD for the Control and AD groups, the statistic provided in the original study was converted by the authors of this review when possible. Values were calculated from raw individual data, estimated from 95% confidence intervals, calculated as weighted means and pooled standard deviations from subgroups, estimated from median and interquartile range using the method of Wan et al., 2014 [20] or calculated from the standard error of the mean, as indicated in Supplementary Table S1A.

### 2.5. Study Design and Validation Classification

To evaluate the study design and validation support underlying each miRNA, we developed a Study Evidence Score (SES), which was assigned to each reporting study. The SES was defined according to predefined and hierarchized categories based on the analytical approach (discovery or targeted analysis) and validation strategy (external or internal cohort and source of sample used for validation), as summarized in Supplementary Figure S1A. The SES was used as a descriptive indicator of study design and validation support. However, it was not intended to represent a formal assessment of methodological quality, risk of bias, or certainty of evidence.

### 2.6. Cross-Study Integration and Consistency Assessment

After generating a table containing all miRNAs that exhibited changes in AD, shared miRNAs between blood and CSF were analyzed for their expression patterns across studies. No conventional pooled effect measure was calculated; instead, shared miRNAs between blood and CSF were compared across studies according to their direction of dysregulation and the number of independent studies supporting each trend. Specifically, we evaluated the number of studies identifying each individual miRNA. Those miRNAs showing consistent differential expression in both blood and CSF were subsequently selected for inclusion in Table 1 and Table 2, where we indicate whether they were consistently increased or decreased in both fluids in the context of AD. Additionally, we summarized miRNAs showing differential expression exclusively in a single biofluid in Table 3.

To quantify the degree of agreement across studies for each miRNA within each source type (blood and CSF), we developed a consistency score (CS). For each source type, the CS was calculated as the absolute difference between the number of studies supporting the predominant direction of dysregulation and the number supporting the opposite direction. When equal numbers of studies reported opposite directions of change within the same fluid, the miRNA was classified as inconsistent. For miRNAs identified in both blood and CSF, a cumulative CS was calculated as the sum of the consistency scores obtained for blood and CSF. This cumulative score reflects within-fluid directional reproducibility across both biofluids and was not intended to measure concordance of direction between blood and CSF. Cross-fluid directionality was assessed separately by classifying miRNAs as showing concordant or discordant expression patterns. The calculation of the CS is illustrated in Supplementary Figure S1B.

**Figure 1:**
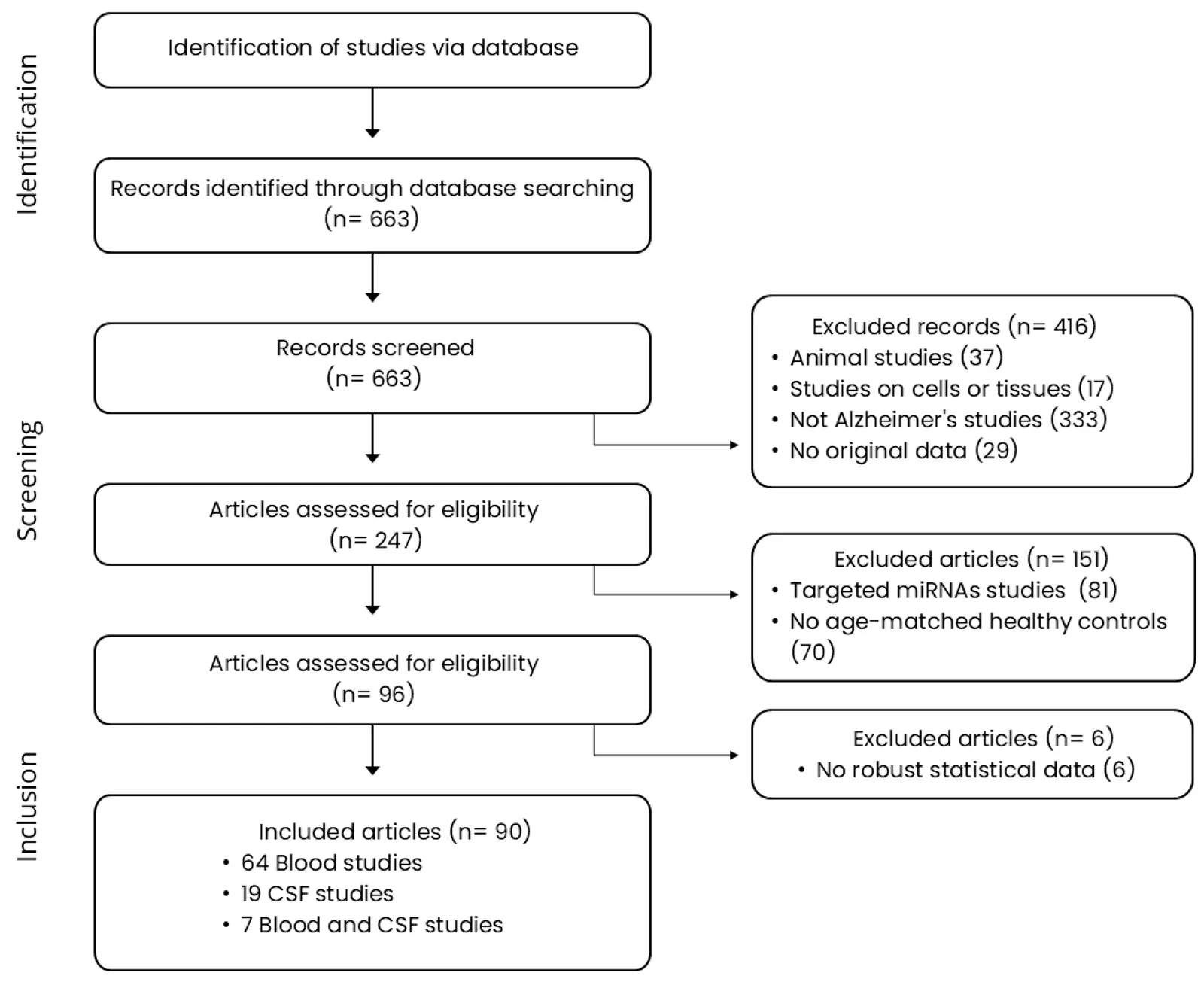
PRISMA flow diagram to select eligible studies.

**Figure 2:**
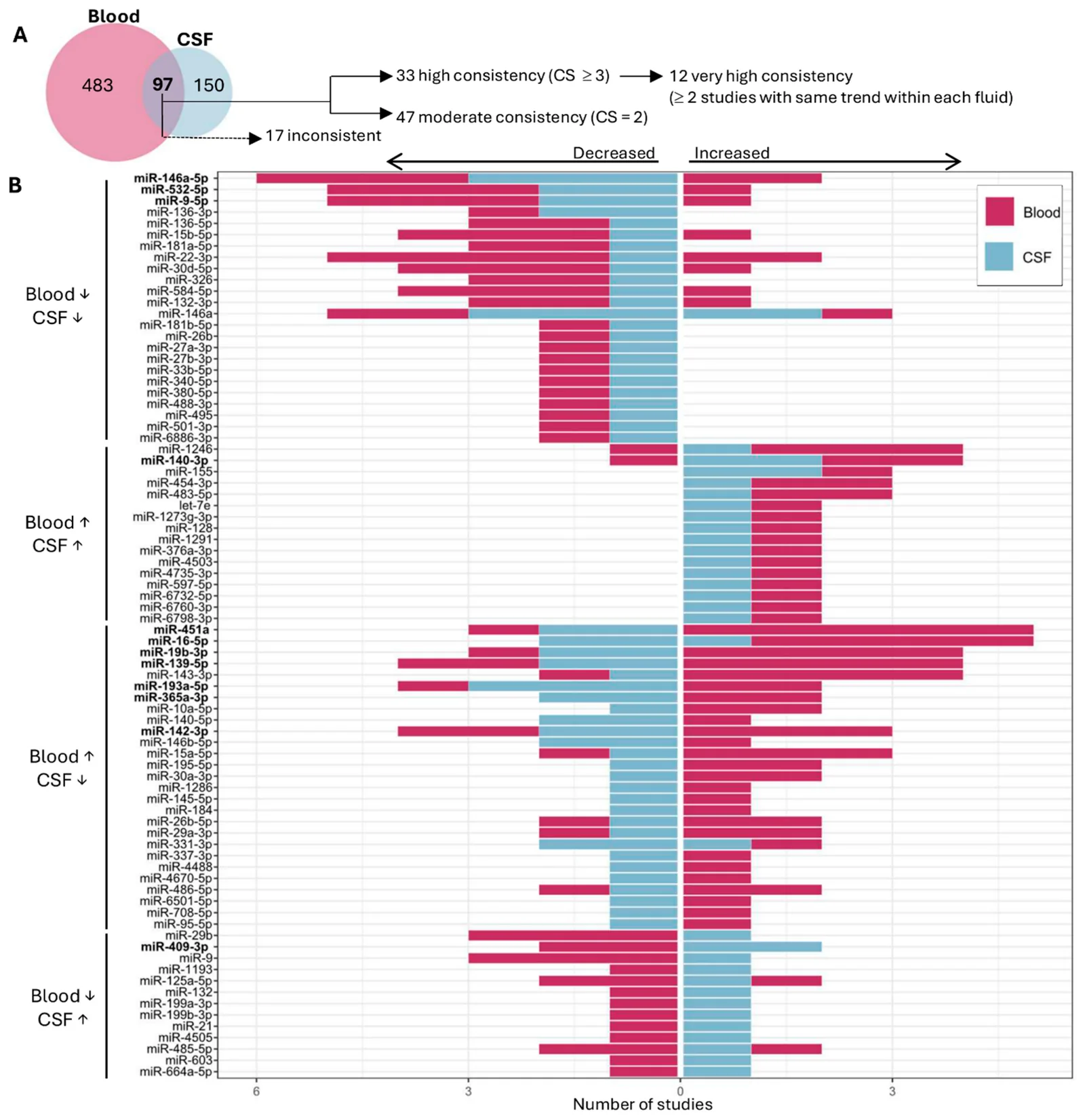
Summary of the miRNAs identified in blood and CSF across the included studies. (**A**) Venn diagram showing the overlap between miRNAs identified in blood and CSF. A total of 80 shared miRNAs were further analyzed after exclusion of those with inconsistent results. (**B**) Number of studies reporting each of the 80 miRNAs and their corresponding trend. The predominant trend in each fluid was used to define the four categories shown for each miRNA. ↑ Upregulated; ↓ Downregulated.

**Figure 3:**
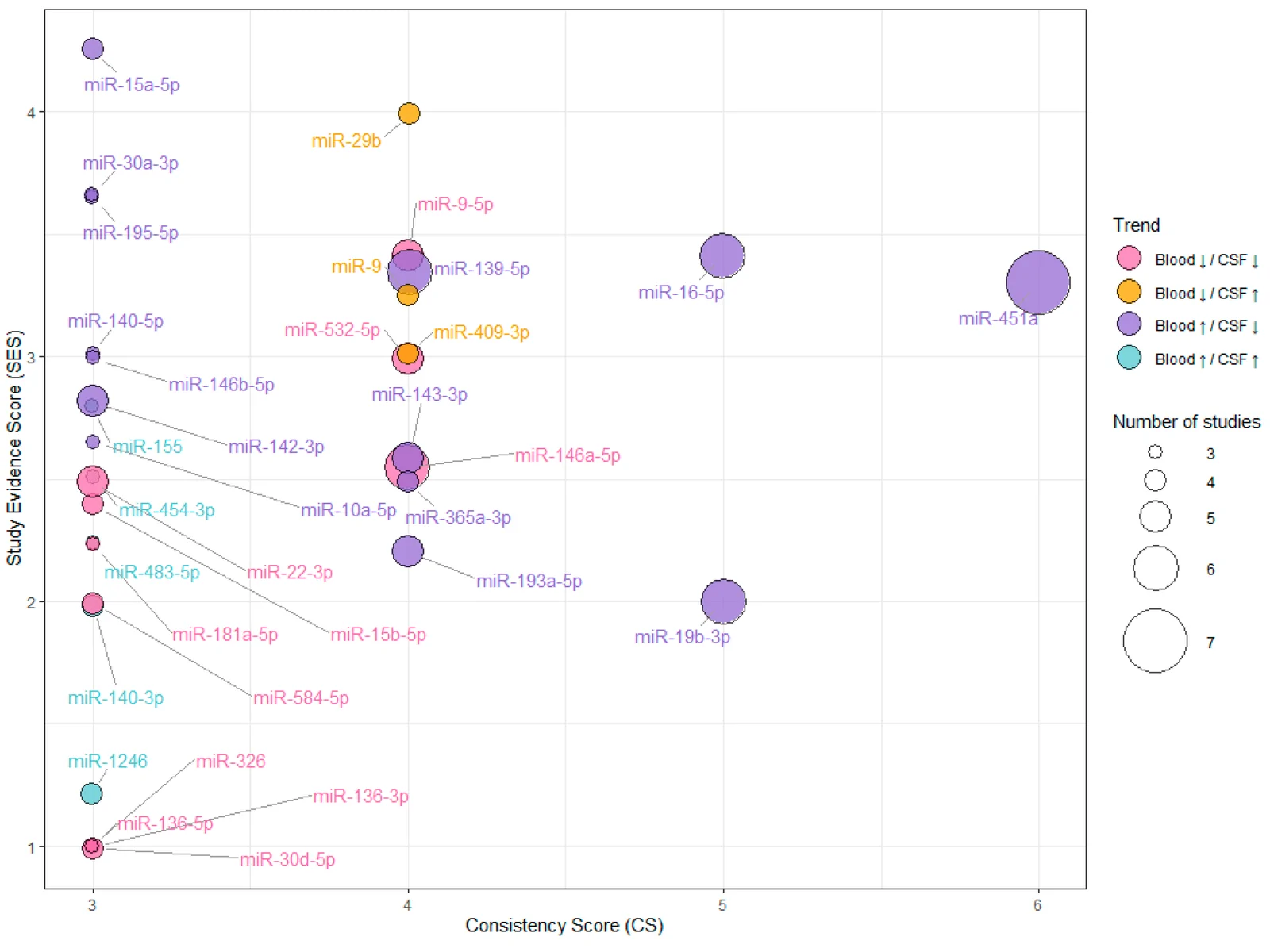
Bubble plot showing high-consistency miRNAs (CS ≥ 3) across blood and CSF according to their cumulative CS and SES. Bubble size is proportional to the total number of studies reporting each miRNA. The color of the bubble indicates the direction of change in the miRNA between fluids.

Results from individual studies and the resulting syntheses were tabulated in Supplementary Table S1A–C and displayed in Table 1, Table 2 and Table 3 and Figure 1, Figure 2 and Figure 3. Given the methodological heterogeneity of the included studies, including differences in sample type (e.g., serum, plasma, whole blood, extracellular vesicles, or blood cells), miRNA detection technology, study design, diagnostic criteria, APOE assessment, and statistical or computational methods, the review used a direction-of-effect and consistency-based synthesis rather than a conventional pooled meta-analysis. For the primary cross-biofluid analysis, blood-derived sample types were grouped under the broader category of “blood”, while the specific sample type used in each study was retained in Supplementary Table S1A. These sources of heterogeneity were documented descriptively rather than explored through meta-regression. No formal sensitivity analyses based on pooled effect estimates were conducted.

### 2.7. Study of Evidence Assessment

To complement the consistency analysis, we characterized the study design and validation support underlying the predominant expression pattern of each miRNA using the Study Evidence Score (SES) described in *Study design and validation classification* and illustrated in Supplementary Figure S1A. For each miRNA, the mean SES was calculated across studies supporting the predominant direction of expression and was interpreted descriptively as an indicator of study design and validation support.

Directional inconsistency was assessed separately using the consistency score (CS), as described in the previous section, and illustrated in Supplementary Figure S1B. The CS incorporates studies reporting both the predominant and opposite directions of change. Thus, CS and mean SES represent complementary dimensions: CS reflects net directional support across independent studies, whereas mean SES characterizes the design and validation support of studies supporting the predominant expression pattern. For miRNAs showing high cumulative CS values (CS ≥ 3), cumulative CS and mean SES were displayed separately in Figure 3.

### 2.8. Registration and Protocol

This systematic review was registered in the International Prospective Register of Systematic Reviews (PROSPERO; registration number CRD420261496077).

### 2.9. Identification of miRNA -Targeted Proteins

To provide biological support for our miRNA findings, we mapped the set of proteins under their regulation using the R package multiMiR (v1.28.0), which integrates multiple databases for miRNA targets. The analysis was performed using MirTarBase (v9.0), a curated and updated repository of experimentally validated miRNA-target interactions, and included only those supported by strong experimental evidence as demonstrated by the platform, such as reporter assays, Western blots, or other direct validation methods. This approach enabled the identification of protein targets for miRNAs observed in both blood and CSF, providing a biologically grounded framework for interpreting our findings.

Proteins regulated by the common miRNAs identified in our study were compiled based on experimentally supported interactions. To enhance robustness and focus on proteins with potential coordinated regulation, only targets regulated by at least three shared miRNAs were included in the final analysis. It is worth noting that the reliability of these associations depends on the available experimental evidence reported in the literature.

### 2.10. Identification of Disease Association

As a final step to link CSF and blood common miRNAs to AD, we investigated their experimentally supported disease associations. To this end, we used the RNA-Disease Repository (v4.0), a curated database of validated RNA-disease interactions with an emphasis on miRNA-disease relationships. Only associations assigned the highest confidence score (score = 1) by the repository developers were included. This approach enabled the identification of links between common miRNAs and AD, as well as other related disease entities.

This systematic review was conducted in accordance with ethical standards and the PRISMA guidelines, ensuring transparency, reproducibility, and methodological rigor.

## 3. Results

### 3.1. Search Strategy

An extensive literature search following the parameters described in the Section 2 yielded a first pool of 663 publications, of which 416 entries were excluded, mostly due to the use of cell or tissue specimens or fluids of non-human origin. The remaining 247 publications were subjected to a second eligibility review. Out of these, 151 studies were excluded since they were not focused on biomarker discovery. These studies either focused exclusively on one or two preselected miRNAs or failed to compare AD and healthy control groups. Finally, an additional set of 6 studies were excluded due to the absence of significant outcomes or lack of robust statistical data. In total, 90 studies were included in the systematic review. Among these, 64 studies examined circulating miRNAs within blood samples, while 19 studies addressed the analysis of miRNAs in CSF. Additionally, 7 studies provided results from both blood and CSF analyses. An overview of the steps underlying this classification is shown in Figure 1.

A complete list of the included studies and all extracted information is provided in Supplementary Table S1A. The final dataset comprised 7602 participants, of whom 3849 had AD and 3753 were age-matched healthy controls. Neuropsychological criteria for the definition of diagnostic groups differed across studies. While the majority of studies adhered to the National Institute on Aging-Alzheimer’s Association (NIA-AA) guidelines, others relied on standard dementia criteria, including the Clinical Dementia Rating (CDR), cognitive assessments such as the Mini-Mental State Examination (MMSE), neurological evaluations, and/or medical histories. Several studies combine multiple diagnostic criteria to enhance diagnostic accuracy, including the integration of biomarker assessments such as amyloid-β and tau levels.

In terms of study design, the most common approach was multiple targeted analyses, followed by discovery studies with external validation and discovery-only studies. In addition, not all studies assessed freely circulating miRNAs; 23 analyzed extracellular vesicle-associated miRNAs and 4 focused on cell-associated miRNAs from blood (Supplementary Figure S2). qRT-PCR was the most frequently used detection method, followed by RNA sequencing and microarray analysis. Because these technologies differ in sensitivity and quantitative output, this methodological variability was documented to characterize the included studies, without affecting the analytical framework of the review.

It should be noted that 21 studies reported APOE genotype information; however, APOE was not consistently analyzed as an effect modifier, and only several studies reported no significant influence of genotype on their miRNA findings. Also, regarding statistical approaches, most studies used standard hypothesis-testing methods, although some relied on regression-based or machine learning approaches.

### 3.2. Consistently Dysregulated miRNAs in CSF and Blood of AD Patients

We analyzed miRNA expression data from each of the 90 publications, extracting miRNAs that were significantly upregulated or downregulated in blood or CSF and their specific comparison. This study identified 730 unique miRNAs altered in AD (Supplementary Table S1B). Among them, 483 were blood-specific, 150 were CSF-specific, and 97 were shared between both biological fluids (Figure 2A). To evaluate the robustness of miRNA dysregulation, we compared the direction of change reported across studies (Figure 2B).

Of those miRNAs identified in both biological fluids, 17 showed low consistency or inconsistent findings, 47 showed moderate consistency (CS = 2), and 33 exhibited high cumulative consistency (CS ≥ 3) (Figure 2). Of the latter, 12 were supported by at least two studies with the same trend within each fluid and were therefore classified as having very high consistency (highlighted in bold in Table 1). A detailed summary of miRNA expression, including those whose CS did not meet the above cutoffs, is provided in Supplementary Table S1C. Within the set of high-consistency miRNAs (CS ≥ 3), 16 showed broadly concordant expression changes across fluids: miR-1246, miR-140-3p, miR-155, miR-454-3p, miR-483-5p were increased in AD, while miR-146a-5p, miR-532-5p, miR-9-5p, miR-136-3p, miR-136-5p, miR-15b-5p, miR-181a-5p, miR-22-3p, miR-30d-5p, miR-326 and miR-584-5p were downregulated (Table 1A). Of them, 4 miRNAs were identified as showing very high cumulative consistency across biological fluids (miR-140-3p, miR-146a-5p, miR-532-5p, and miR-9-5p). Conversely, 17 miRNAs exhibited discordant trends between blood and CSF. miR-451a, miR-16-5p, miR-19b-3p, miR-139-5p, miR-143-3p, miR-193a-5p, miR-365a-3p, miR-10a-5p, miR-140-5p, miR-142-3p, miR-146b-5p, miR-15a-5p, miR-195-5p, miR-30a-3p were found to be increased in blood and decreased in CSF, while miR-29b, miR-409-3p, miR-9 were increased in CSF and decreased in blood (Table 1B). Similarly, 8 miRNAs exhibited the highest consistency across biological fluids (miR-451a, miR-16-5p, miR-19b-3p, miR-139-5p, miR-193a-5p, miR-365a-3p, miR-142-3p, and miR-409-3p).

**Table 1:** Similar and Opposite miRNA signatures across blood and CSF in Alzheimer’s disease having consistency score (CS) ≥ 3. Regulation patterns are indicated as the same trend (**A**) or opposite trend (**B**) in both fluids across independent studies, together with the number of consistent studies reporting each miRNA. miRNAs labeled with an asterisk (*) were identified in both CSF and blood within the same study. ↑ Upregulated miRNAs; ↓ Downregulated miRNAs. miRNAs highlighted in bold were considered to show very high consistency (identified in at least two studies with the same trend within each fluid).

miRNA	Blood AD		CSF AD		Trend	CS
	↑	↓	↑	↓	Blood/CSF	
(A) miRNAs altered in blood and CSF (Same trend)						
*Increase*						
miR-1246	3	1	1	0	↑/↑	3
**miR-140-3p**	2	1	2	0	↑/↑	3
miR-155	1	0	2	0	↑/↑	3
miR-454-3p	2	0	1	0	↑/↑	3
miR-483-5p	2	0	1	0	↑/↑	3
*Decrease*						
**miR-146a-5p**	2	3	0	3	↓/↓	4
**miR-532-5p**	1	3	0	2	↓/↓	4
**miR-9-5p**	1	3	0	2	↓/↓	4
miR-136-3p	0	1	0	2	↓/↓	3
miR-136-5p	0	2	0	1	↓/↓	3
miR-15b-5p	1	3	0	1	↓/↓	3
miR-181a-5p	0	2	0	1	↓/↓	3
miR-22-3p	2	4	0	1	↓/↓	3
miR-30d-5p	1	3	0	1	↓/↓	3
miR-326	0	2	0	1	↓/↓	3
miR-584-5p	1	3	0	1	↓/↓	3
(B) miRNAs altered in blood and CSF (Opposite trend)						
*Increase/decrease*						
**miR-451a**	5	1	0	2	↑/↓	6
**miR-16-5p**	4	0	1	2	↑/↓	5
**miR-19b-3p**	4	1	0	2	↑/↓	5
**miR-139-5p**	4	2	0	2	↑/↓	4
miR-143-3p	4	1	0	1	↑/↓	4
**miR-193a-5p**	2	1	0	3	↑/↓	4
**miR-365a-3p**	2	0	0	2	↑/↓	4
miR-10a-5p	2	0	0	1	↑/↓	3
miR-140-5p	1	0	0	2	↑/↓	3
**miR-142-3p**	3	2	0	2	↑/↓	3
miR-146b-5p	1	0	0	2	↑/↓	3
miR-15a-5p *	3	1	0	1	↑/↓	3
miR-195-5p	2	0	0	1	↑/↓	3
miR-30a-3p	2	0	0	1	↑/↓	3
*Decrease/increase*						
miR-29b	0	3	1	0	↓/↑	4
**miR-409-3p**	0	2	2	0	↓/↑	4
miR-9	0	3	1	0	↓/↑	4

To further characterize the evidence supporting the high-consistency miRNAs, directional reproducibility and study design/validation support were examined as complementary dimensions (Figure 3). miR-451a and miR-16-5p showed the highest cumulative consistency (CS = 6 and 5, respectively), together with intermediate mean SES values. miR-19b-3p also showed high directional consistency (CS = 5), but a lower mean SES, reflecting that most studies supporting its predominant expression pattern were discovery studies without additional validation. Conversely, miR-15a-5p showed lower cumulative consistency (CS = 3) but the highest mean SES, indicating that the studies supporting its predominant expression pattern included a greater degree of validation. These examples illustrate that directional reproducibility and study design/validation support provide complementary information and do not necessarily increase in parallel.

Additionally, 47 miRNAs were classified as moderately consistent, as they achieved a CS = 2, corresponding to a score of 1 in each fluid (Table 2). Among them, some showed concordant patterns between fluids. Specifically, let-7e, miR-1273g-3p, miR-128, miR-1291, miR-376a-3p, miR-4503, miR-4735-3p, miR-597-5p, miR-6732-5p, miR-6760-3p, miR-6798-3p were found to be upregulated (Table 2A), and miR-132-3p, miR-146a, miR-181b-5p, miR-26b, miR-27a-3p, miR-27b-3p, miR-33b-5p, miR-340-5p, miR-380-5p, miR-488-3p, miR-495, miR-501-3p, miR-6886-3p were downregulated in both fluids of AD patients (Table 2B). In contrast, others, despite being detected in both fluids, exhibited opposite expression patterns between them (Table 2C). Whereas miR-1286, miR-145-5p, miR-184, miR-26b-5p, miR-29a-3p, miR-331-3p, miR-337-3p, miR-4488, miR-4670-5p, miR-486-5p, miR-6501-5p, miR-708-5p, miR-95-5p were upregulated in blood but downregulated in CSF, miR-1193, miR-125a-5p, miR-132, miR-199a-3p, miR-199b-3p, miR-21, miR-4505, miR-485-5p, miR-603, miR-664a-5p were downregulated in blood and upregulated in CSF.

**Table 2:** Consistent and Opposite miRNA signatures across Blood and CSF in Alzheimer’s disease having a consistency score (CS) ≥ 2. Regulation patterns are indicated as the same trend (**A**,**B**) or opposite trend (**C**) in both fluids across independent studies, together with the number of consistent studies reporting each miRNA. miRNAs marked with an asterisk (*) were identified in both CSF and blood within the same study. ↑ Upregulated miRNAs; ↓ Downregulated miRNAs.

miRNA	Blood AD		CSF AD		Trend	CS
	↑	↓	↑	↓	Blood/CSF	
(A) miRNAs altered in blood and CSF (at least 1 study per fluid and same trend UP)						
let-7e	1	0	1	0	↑/↑	2
miR-1273g-3p *	1	0	1	0	↑/↑	2
miR-128	1	0	1	0	↑/↑	2
miR-1291	1	0	1	0	↑/↑	2
miR-376a-3p	1	0	1	0	↑/↑	2
miR-4503	1	0	1	0	↑/↑	2
miR-4735-3p *	1	0	1	0	↑/↑	2
miR-597-5p	1	0	1	0	↑/↑	2
miR-6732-5p	1	0	1	0	↑/↑	2
miR-6760-3p *	1	0	1	0	↑/↑	2
miR-6798-3p *	1	0	1	0	↑/↑	2
(B) miRNAs altered in blood and CSF (at least 1 study per fluid and same trend DOWN)						
miR-132-3p	1	2	0	1	↓/↓	2
miR-146a	1	2	2	3	↓/↓	2
miR-181b-5p	0	1	0	1	↓/↓	2
miR-26b	0	1	0	1	↓/↓	2
miR-27a-3p	0	1	0	1	↓/↓	2
miR-27b-3p	0	1	0	1	↓/↓	2
miR-33b-5p	0	1	0	1	↓/↓	2
miR-340-5p	0	1	0	1	↓/↓	2
miR-380-5p	0	1	0	1	↓/↓	2
miR-488-3p	0	1	0	1	↓/↓	2
miR-495	0	1	0	1	↓/↓	2
miR-501-3p	0	1	0	1	↓/↓	2
miR-6886-3p	0	1	0	1	↓/↓	2
(C) miRNAs altered in blood and CSF (at least 1 study per fluid and oposite trend)						
*Increase/decrease*						
miR-1286 *	1	0	0	1	↑/↓	2
miR-145-5p	1	0	0	1	↑/↓	2
miR-184	1	0	0	1	↑/↓	2
miR-26b-5p	2	1	0	1	↑/↓	2
miR-29a-3p	2	1	0	1	↑/↓	2
miR-331-3p	1	0	1	2	↑/↓	2
miR-337-3p	1	0	0	1	↑/↓	2
miR-4488	1	0	0	1	↑/↓	2
miR-4670-5p *	1	0	0	1	↑/↓	2
miR-486-5p	2	1	0	1	↑/↓	2
miR-6501-5p	1	0	0	1	↑/↓	2
miR-708-5p	1	0	0	1	↑/↓	2
miR-95-5p *	1	0	0	1	↑/↓	2
*Decrease/increase*						
miR-1193	0	1	1	0	↓/↑	2
miR-132	0	1	1	0	↓/↑	2
miR-199a-3p	0	1	1	0	↓/↑	2
miR-199b-3p	0	1	1	0	↓/↑	2
miR-21	0	1	1	0	↓/↑	2
miR-125a-5p	1	2	1	1	↓/↑	2
miR-4505 *	0	1	1	0	↓/↑	2
miR-485-5p	1	2	1	0	↓/↑	2
miR-603	0	1	1	0	↓/↑	2
miR-664a-5p	0	1	1	0	↓/↑	2

### 3.3. Fluid-Specific Dysregulated miRNAs in CSF and Blood of AD Patients

To further characterize fluid-specific patterns, we analyzed miRNAs identified exclusively in blood or CSF. Of the 483 blood-specific miRNAs (i.e., not identified in CSF studies), 27 showed inconsistent results, with an equal number of studies reporting opposite directions of change, whereas 385 showed low consistency (CS = 1) (Supplementary Table S1C). The remaining 71 miRNAs, together with four additional miRNAs that were also reported in CSF studies but showed inconsistent results in that source (miR-223-3p, miR-378a-3p, miR-125b-5p and miR-34a), were classified in our analysis as exclusively dysregulated in blood across AD studies (Table 3). Of these, 31 were consistently reported as upregulated (Table 3A), while 44 were consistently reported as downregulated (Table 3B). The most consistently upregulated miRNAs were miR-223-3p and miR-424-5p whereas the most consistently downregulated were let-7d-5p, miR-107, miR-342-3p, let-7a-5p, let-7i-5p, miR-103a-3p, and miR-221-3p; with a CS ≥ 4.

**Table 3:** miRNAs consistently altered and exclusively identified in Blood (**A**,**B**) or CSF (**C**,**D**). miRNAs identified that showed upregulation (**A**,**C**) or downregulation (**B**,**D**) in at least two independent studies. Within the same fluid, miRNAs for which opposite trends are reported in the same number of independent studies are denoted as inconsistent; therefore, their global trend is not counted. Total consistent studies are calculated as the difference between the number of studies reporting the corresponding trend and the number of studies reporting the opposite trend in the same fluid. Only miRNAs for which total consistent studies are ≥2 are shown. ↑ Upregulated miRNAs; ↓ Downregulated miRNAs.

miRNA	Blood AD		CSF AD		Trend	CS
	↑	↓	↑	↓	Blood/CSF	
(A) miRNAs altered exclusively in blood (≥2 studies-UP)						
miR-223-3p	6	1	1	1	↑/-	5
miR-424-5p	5	0	0	0	↑/-	5
miR-183-5p	3	0	0	0	↑/-	3
miR-20a-5p	5	2	0	0	↑/-	3
miR-363-3p	3	0	0	0	↑/-	3
miR-5006-3p	3	0	0	0	↑/-	3
miR-93-5p	5	2	0	0	↑/-	3
let-7d-3p	3	1	0	0	↑/-	2
miR-10b-5p	2	0	0	0	↑/-	2
miR-122-5p	2	0	0	0	↑/-	2
miR-125b-1-3p	2	0	0	0	↑/-	2
miR-1285-3p	2	0	0	0	↑/-	2
miR-1285-5p	2	0	0	0	↑/-	2
miR-1-3p	2	0	0	0	↑/-	2
miR-1468-5p	2	0	0	0	↑/-	2
miR-18b-5p	2	0	0	0	↑/-	2
miR-192-5p	2	0	0	0	↑/-	2
miR-20b-5p	3	1	0	0	↑/-	2
miR-335-5p	2	0	0	0	↑/-	2
miR-345-5p	3	1	0	0	↑/-	2
miR-34b-5p	2	0	0	0	↑/-	2
miR-361-5p	3	1	0	0	↑/-	2
miR-378a-3p	3	1	1	1	↑/-	2
miR-425-5p	3	1	0	0	↑/-	2
miR-4732-3p	2	0	0	0	↑/-	2
miR-486-3p	2	0	0	0	↑/-	2
miR-5001-3p	2	0	0	0	↑/-	2
miR-5010-3p	2	0	0	0	↑/-	2
miR-502-3p	2	0	0	0	↑/-	2
miR-548at-5p	2	0	0	0	↑/-	2
miR-7-1-3p	2	0	0	0	↑/-	2
(B) miRNAs altered exclusively in blood (≥2 studies-DOWN)						
let-7d-5p	0	5	0	0	↓/-	5
miR-107	0	5	0	0	↓/-	5
miR-342-3p	1	6	0	0	↓/-	5
let-7a-5p	0	4	0	0	↓/-	4
let-7i-5p	0	4	0	0	↓/-	4
miR-103a-3p	1	5	0	0	↓/-	4
miR-221-3p	0	4	0	0	↓/-	4
miR-125b-5p	1	4	1	1	↓/-	3
miR-1306-5p	0	3	0	0	↓/-	3
miR-130b-3p	0	3	0	0	↓/-	3
miR-151a-3p	2	5	0	0	↓/-	3
miR-191-5p	0	3	0	0	↓/-	3
miR-21-5p	1	4	0	0	↓/-	3
miR-320a	0	3	0	0	↓/-	3
miR-338-3p	0	3	0	0	↓/-	3
miR-423-3p	0	3	0	0	↓/-	3
miR-4433b-5p	0	3	0	0	↓/-	3
miR-92a-3p	2	5	0	0	↓/-	3
miR-15b-3p	0	2	0	0	↓/-	2
let-7c-5p	0	2	0	0	↓/-	2
miR-122	0	2	0	0	↓/-	2
miR-126-5p	0	2	0	0	↓/-	2
miR-1307-3p	0	2	0	0	↓/-	2
miR-130a-3p	0	2	0	0	↓/-	2
miR-150-5p	1	3	0	0	↓/-	2
miR-152-3p	0	2	0	0	↓/-	2
miR-17-3p	0	2	0	0	↓/-	2
miR-17-5p	0	2	0	0	↓/-	2
miR-197-3p	0	2	0	0	↓/-	2
miR-23b-3p	1	3	0	0	↓/-	2
miR-34a	0	2	1	1	↓/-	2
miR-34a-5p	0	2	0	0	↓/-	2
miR-381-3p	0	2	0	0	↓/-	2
miR-382-5p	0	2	0	0	↓/-	2
miR-425-3p	0	2	0	0	↓/-	2
miR-432-5p	0	2	0	0	↓/-	2
miR-4419a	0	2	0	0	↓/-	2
miR-4433b-3p	0	2	0	0	↓/-	2
miR-548k	0	2	0	0	↓/-	2
miR-574-5p	0	2	0	0	↓/-	2
miR-628-3p	0	2	0	0	↓/-	2
miR-654-5p	0	2	0	0	↓/-	2
miR-744-5p	0	2	0	0	↓/-	2
miR-98-5p	0	2	0	0	↓/-	2
(C) miRNAs altered exclusively in CSF (≥2 studies-UP)						
miR-29a	1	1	2	0	-/↑	2
miR-30a-5p	0	0	2	0	-/↑	2
miR-484	1	1	2	0	-/↑	2
(D) miRNAs altered exclusively in CSF (≥2 studies-DOWN)						
miR-3200-3p	0	0	0	2	-/↓	2
miR-598	0	0	0	2	-/↓	2

In contrast, most miRNAs identified exclusively in CSF showed very low consistency. Of the 150 CSF-specific miRNAs, 146 had a consistency score of 1, while one miRNA, miR-590-5p, had a CS of 0, reflecting opposite directions of change across independent studies. The remaining two miRNAs, together with two additional miRNAs that were also identified in blood but showed a CS of 0 in that source (miR-29a and miR-484), were classified in our analysis as exclusively dysregulated in AD CSF (Table 3C,D). Among these, miR-29a, miR-30a-5p, and miR-484 were consistently upregulated, whereas miR-3200-3p and miR-598 were consistently downregulated (Table 3C,D).

### 3.4. miRNA Target Analysis

To explore the biological relevance of the 33 miRNAs exhibiting high consistency across studies (CS ≥ 3) in CSF and blood (Table 1), we examined experimentally validated miRNA-target interactions using MirTarBase as described in Section 2. Although 4 miRNAs had no validated protein targets (miR-125b, miR-155, miR-29b, and miR-9 were reported in precursor rather than mature forms and lacked sufficient experimental validation), this analysis revealed overlapping targets among the miRNAs identified. Complete data on the targets of all meta-analyzed miRNAs are summarized in Supplementary Table S2.

**Figure 4:**
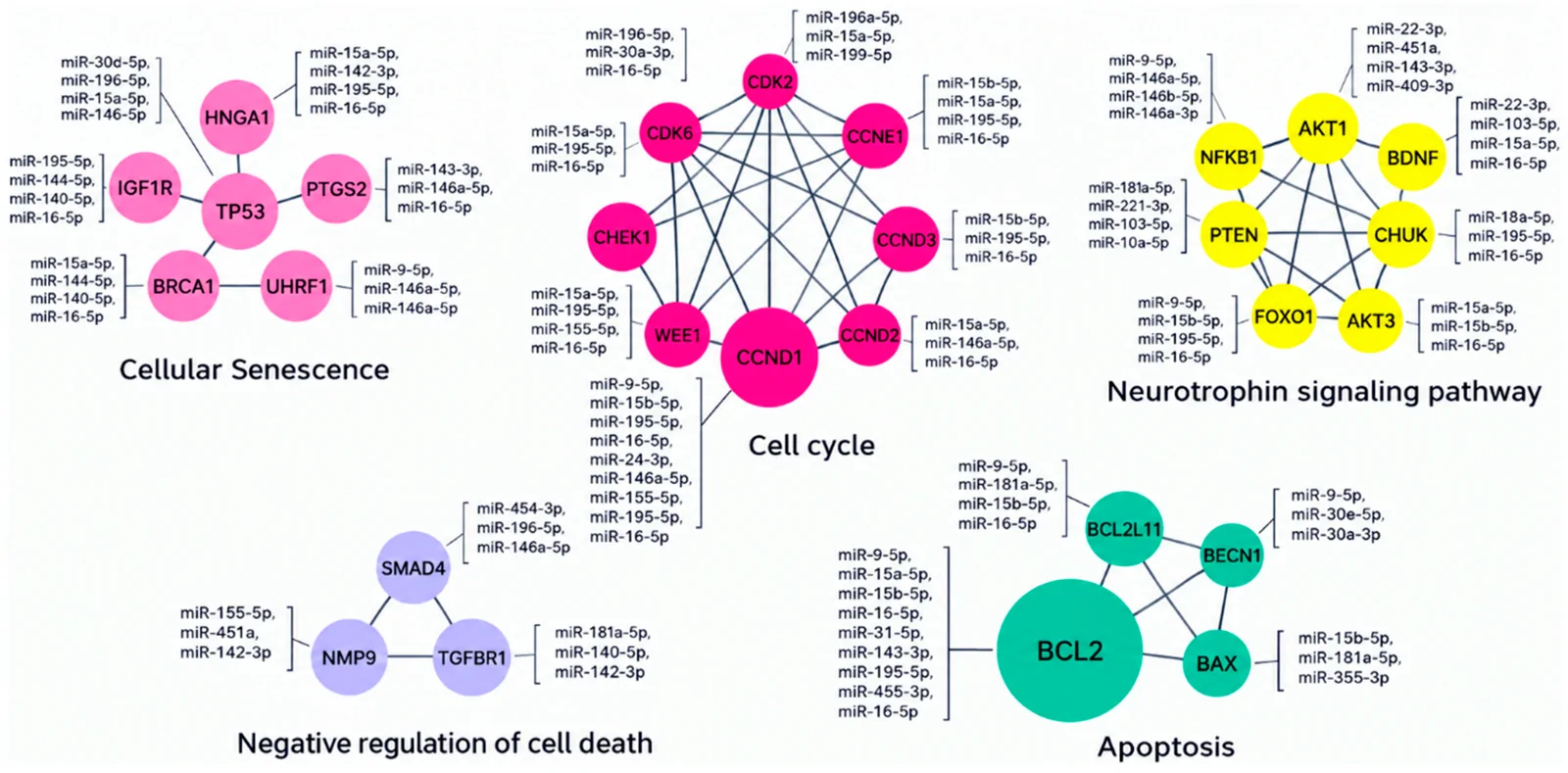
Schematic diagram of miRNAs identified in blood and CSF and their role in AD pathogenesis. The figure shows the main biological pathways and functional modules associated with the 48 proteins most robustly regulated by the 33 miRNAs exhibiting high consistency across studies (CS ≥ 3). Pathways are grouped into apoptosis, cell cycle-related processes, and neurotrophin signaling pathways. Node size reflects the number of miRNAs associated with each protein, and edges indicate shared miRNA regulation among target proteins.

For the functional analysis, we focused exclusively on the 33 miRNAs exhibiting high consistency across studies (CS ≥ 3), resulting in 48 proteins most robustly associated with these miRNAs. Among them, BCL2, CCND1, IGF1R, AKT1, BCL2L11, WEE1, TP53, CCNE1, BDNF, NFKB1, CHEK1, PTEN and HMGA1 were regulated by 4 or more miRNA, while the proteins FOXO1, CDK6, CCND2, PTGS2, MMP9 AKT3, BECN1, UHRF1, CCND3, BAX, CDC25A, BRCA1, SMAD4, TGFBR1 and CHUK were targeted by 3 different miRNA. The most represented biological pathways included cell-cycle regulation, neuronal signaling and glial differentiation, tissue remodeling, cell death, and cellular senescence (Figure 4). When we explored the regulatory miRNAs, we observed that most of them corresponded to those that were upregulated in blood and downregulated in CSF (Supplementary Table S2).

### 3.5. miRNA-Disease Associations

We further examined potential associations between the 33 common miRNAs and other diseases using the “RNA-Disease Repository”. Each miRNA was associated with multiple diseases (Supplementary Table S3). Together with different types of cancer, the most representative disease was AD (Supplementary Table S3, highlighted in gray) which was associated with 11 miRNAs (miR-483-5p, miR-146a-5p, miR-9-5p, miR-15b-5p, miR-22-3p, miR-16-5p, miR-19b-3p, miR-143-3p, miR-142-3p, miR-15a-5p and miR-30a-3p). These findings open new avenues for exploring the potential role of additional miRNAs that have not yet been associated with this pathology.

## 4. Discussion

Comparison of miRNAs across different studies within blood and CSF samples has been proven by different systematic reviews to be a difficult task given the heterogeneous methodology used for miRNA detection [21,22]. There are several studies conducted in blood [23,24,25,26] that have identified systemic miRNA alterations, providing valuable information on the potential of circulating miRNAs as accessible and minimally invasive biomarkers for AD diagnosis. Alternatively, other studies have identified several miRNAs in CSF [15,27,28,29,30] to provide complementary information that more accurately reflects CNS alterations, thus enhancing the biological relevance of neuropathological changes. Our multi-comparative study compiles information on study design, experimental approaches, the biological fluid in which each miRNA is detected (blood or CSF), and their pattern of change in AD based exclusively on results supported by strong statistical evidence in order to build a robust and reliable database. This novel approach has allowed us to propose the most valuable similarities and differences in the behavior of miRNAs in relation to AD across both fluids.

Even though we started with 663 records, we ended up including 90 studies, primarily due to the absence of direct comparison between AD and age-matched healthy control groups or a robust statistical analysis [30,31]. Further exclusions were imposed on studies employing non-human samples [32], cellular or tissue models [33], or studies exclusively assessing one or two preselected candidate miRNAs, which fell outside the biomarker-discovery and multi-candidate profiling scope established for this review [34,35].

Our analysis identified 33 miRNAs that exhibited consistent dysregulation in blood and CSF in AD compared with cognitively normal controls, 12 of which were reported in at least two studies per fluid. Detection of these miRNAs in both fluids underscores their robustness and their relevance as accessible indicators of AD-associated neuropathological processes. Indeed, our study unveils 8 novel miRNAs that were not reported in previously published meta-analyses. Five of them follow the same direction of change in both fluids, while three are upregulated in blood and downregulated in CSF.

### 4.1. miRNAs with Shared Dysregulation in Blood and CSF in AD

Our analysis is consistent with previous meta-analyses, identifying several miRNAs previously reported as major factors in AD, including miR-1246, miR-155 and miR-483-5p [36,37,38,39], which emerged among the most consistently upregulated in both blood and CSF. While at first they appeared to be robust candidates, all have been described previously as dysregulated not only in AD [18,24,40,41,42] but also in other neurodegenerative diseases such as Amyotrophic Lateral Sclerosis [43,44], other pathologies that may affect the brain [45], including intracranial aneurysms [46], glioblastoma (GBM) [47], or even multiple cardiovascular diseases [48,49,50]. Interestingly, whereas circulating miR-155 has been strongly associated with neuroinflammatory processes and the development and progression of neurological disorders [51], our study is the first to report its alteration in blood from AD subjects. Overall, these findings suggest that these miRNAs may be associated with shared pathological processes across diverse conditions. Alternatively, our study provides the first robust evidence that miR-140-3p and miR-454-3p are both consistently upregulated in CSF and plasma of AD patients. Whereas results for miR-140-3p levels in blood showed some heterogeneity, with one study reporting findings opposite to the general trend, data for miR-454-3p were consistently concordant across studies. miR-140-3p has been associated with cognitive function in the elderly population [52,53], and miR-454-3p has been reported to play diverse roles in various cancers, involving distinct mechanisms in each tumor type [54,55]. Given that they have not been previously linked to the disease, our findings are particularly noteworthy and highlight the need for further studies to clarify the biological relevance of their increased expression, with miR-454-3p being of particular significance due to its regulation of SMAD4 [56], which has been recently implicated in AD pathogenesis [57].

Among the miRNAs downregulated in both blood and CSF, miR-146a-5p, miR-532-5p and miR-9-5p are the most consistent in our study. Despite the fact that studies in humans have reported conflicting results regarding the expression of miR-146a in blood and CSF [58,59], there is general agreement that miR-146a may be involved in the onset of AD, particularly in late-stage disease [28]. Interestingly, miR-9-5p has yielded conflicting results in cellular models of AD, which could be attributed to its dual role in protecting against mitochondrial damage and oxidative stress, as well as its involvement in BACE1 regulation and autophagy modulation [60,61,62].

In contrast, our findings point to miR-532-5p as a novel potential biomarker for AD in human fluids. This miRNA has been associated with improved BBB function and Aβ clearance in the 5XFAD mouse model of AD in response to long-term exercise, potentially mediated by exosome-dependent mechanisms [63]; however, evidence for this marker in human blood or CSF is currently lacking. In this context, other novel candidate biomarkers identified in our study showing consistently decreased levels in human biofluids include miR-30d-5p and miR-584-5p. Both miRNAs have been associated with various human tumor types, including breast, lung, colorectal, prostate, liver, esophageal, and cervical cancers, as well as other types of human tumors [64,65], where miR-30d-5p exerts complex effects on tumor progression and key cellular processes, such as cell proliferation, motility, apoptosis, or autophagy [64], processes which may overlap with alterations observed in AD. Given the current lack of evidence for these markers in AD, they emerge as compelling targets for further investigation.

Our study also highlights a consistent pattern of inverse expression between peripheral and central samples, such as miR-451a or miR-29b. Notwithstanding this, several studies have also reported significant differential expression between blood and various brain tissues, such as miR-10a-5p or miR-195-5p, as reviewed by [36,37,66,67]. These changes in the concentration of miRNAs between tissues and plasma might be explained by a cellular selective mechanism of releasing miRNAs [68].

The most consistent outcome across our studies is miR-451a. The special relevance of this finding lies in several facts. First, there is an opposite expression pattern between blood and CSF; second, it showed the strongest support in our IES-based ranking; and finally, it has been proposed to be among the three most abundant miRNAs in blood (in both serum and plasma) [69,70]. While earlier evidence was limited and inconclusive, more recent findings [37,39], including our own, support the suitability of this miRNA as a potential biomarker. Despite these promising results, they should be interpreted with caution, as hemolysis has been reported to increase the levels of this miRNA, which may be influenced by long-term storage conditions [39,71]. In contrast, to our knowledge, this is the first study to provide robust evidence supporting the dysregulation of miR-16-5p, miR-19b-3p, miR-193a-5p, and miR-142-3p across both fluids. While previous studies have reported altered levels in blood [25,72,73], findings in CSF have been limited and inconsistent [37,39]. This is of particular interest for miR-16-5p, which showed both very high consistency and strong support from studies reporting the predominant trend.

Additional novel findings of our study include miR-365a-3p, miR-140-5p, and miR-146b-5p. Even though they have not been evaluated in AD models, both miRNAs directly target the disintegrin and metalloproteinase domain-containing protein 10 (ADAM10) [74,75], whose expression is dysregulated in AD [76]. In our analysis, miR-365a-3p and miR-140-5p were decreased in CSF, which is consistent with the findings of Vanatabe et al., 2021 [77], who reported elevated ADAM10 levels in CSF of patients with mild AD. By contrast, our results also indicated increased blood levels of these miRNAs, whereas according to [77], increased ADAM10 was found in the plasma of these patients. Besides ADAM10, miR-365a-3p has been proposed to directly target MyD88, inhibiting its expression and downstream NF-κB activation [78]. Therefore, reduced levels of miR-365a-3p in CSF could also be associated with AD-related neuroinflammation, highlighting its potential as a promising biomarker. Of particular relevance is miR-146b-5p, which has been characterized as a relevant diagnostic marker for thyroid carcinomas [79,80], and direct evidence in biofluids from AD patients remains limited and warrants further investigation. Given its established role as a negative regulator of the TLR/NF-κB pathway [81,82], the reduced CSF levels observed in our study may contribute to AD-associated neuroinflammation.

The opposite expression pattern observed for certain miRNAs, such as miR-29b, miR-409-3p and miR-9, which are increased in CSF but reduced in blood, suggests a different underlying mechanism compared with other dysregulated miRNAs. Given that miRNAs in CSF primarily reflect brain expression, this emphasizes the need to consider blood and CSF separately. In addition, BBB integrity is compromised [83], which may also allow greater leakage of miRNAs from the CNS to the periphery. This could reduce their concentration in CSF while increasing their presence in plasma, possibly through altered transporters or vascular inflammation. In blood, they could be upregulated from peripheral sources like activated immune cells (monocytes, lymphocytes) driven by systemic inflammation in AD, or compensatory responses [84]. Additionally, the concentration of several miRNAs in exosomes, whose secretion may be altered in the context of AD [85], could contribute to dissimilar concentrations of miRNAs in blood and CSF. Specifically, miR-29b is one of the most extensively studied miRNAs in AD, and it is directly linked to amyloid pathology through the regulation of BACE1 and Aβ42 expression levels [86,87]. While reduced expression has been reported in brain tissue [86,88], evidence in circulating biofluids remained limited and inconsistent. Therefore, our findings may provide new insights into its potential role as a biomarker detectable across different biofluids.

Although several studies reported no apparent influence of APOE genotype on miRNA findings, this should not be interpreted as evidence that circulating or CSF miRNA profiles are independent of APOE status. The absence of significant associations may partly reflect limited statistical power for genotype-stratified analyses and methodological heterogeneity among studies. Moreover, APOE-related differences in CSF extracellular-vesicle miRNA profiles have also been reported [29], suggesting that these effects may be context- and miRNA-specific. Larger studies specifically designed for APOE-stratified analyses are therefore needed.

### 4.2. Specific Blood or CSF Biomarkers

In addition to the identification of shared miRNAs between blood and CSF, we have also evaluated both fluids independently. The top miRNAs overexpressed exclusively in blood appear to be miR-223-3p, miR-424-5p, miR-183-5p, miR-20a-5p, miR-363-3p, miR-5006-3p and miR-93-5p.

While some of these miRNAs, such as miR-223-3p, miR-424-5p, miR-183-5p and miR-93-5p, have already been proposed as circulating biomarkers of AD based on their involvement in key pathological mechanisms including inflammation [89], BBB permeability [90] or PSEN2 expression [91], our study identifies 3 novel candidate biomarkers that showed increased levels in blood. These miRNAs include miR-20a-5p, which has exclusively been associated with cancer [92], and miR-363-3p and miR-5006-3p, which have been associated with mTOR signaling and cell proliferation [93,94,95], reinforcing their potential involvement in autophagy-related pathways relevant to AD, as discussed further below.

We found herein that different members of the let-7 group of miRNAs were downregulated specifically in the blood of AD patients. The let-7 group of miRNAs has been proposed to contribute to the advancement of AD since fluctuations in the levels of these miRNAs can play a part in the deterioration of neurons, which is a central feature in AD [96]. Although in the present study we did not detect members of the Let-7 miRNA family in CSF, let-7a-5p, let-7d-5p, and let-7i-5p emerged as consistently downregulated in the blood of AD patients. A previous in vitro study demonstrated that overexpression of let-7a in neuronal cells aggravates β-amyloid-induced neurotoxicity, promoting apoptosis and autophagy dysregulation [97]. Importantly, this upregulation was experimentally induced rather than observed in patients. In contrast, let-7a-5p and let-7d-5p were found to be downregulated in the blood of AD patients in our review, highlighting a divergence between peripheral and central patterns of regulation. This apparent contradiction likely reflects compartment-specific dynamics, where circulating miRNA levels may not necessarily mirror neuronal expression and may be influenced by differences in release, clearance, or tissue origin.

Our results also support previous findings showing a decreased expression of miR-107 in AD brain neocortex [98], similar to what is observed in blood, where it is also among the most repressed miRNAs. Similarly, while high levels of miR-191-5p may have some protective effect by inhibiting tau phosphorylation, Aβ secretion, and neuronal cell death [99], low levels may reflect AD progression.

In contrast to blood, our analysis highlights several miRNAs that were exclusively dysregulated in CSF, suggesting a compartment-specific signature that may more directly reflect CNS pathology. miR-29a, miR-30a-5p, and miR-484 were found to be the most consistently upregulated CSF-specific biomarkers for AD, whereas miR-3200-3p and miR-598 emerged as the leading CSF biomarkers with reduced levels. While reduced levels of miR-29a in brain tissue have been associated with increased Aβ production through upregulation of BACE1 expression [86], we observed increased levels in CSF, which are in agreement with previous studies [100]. This apparent discrepancy may reflect the release of miRNAs from affected brain cells into the CSF. In addition, miR-30a-5p has been observed to promote Aβ_1-42_ overproduction by inhibiting the nonamyloidogenic pathway by downregulating ADAM10 and SIRT1 [101]. Notably, miR-484 and miR-3200-3p have primarily been described as dysregulated in blood [102,103], while miR-598 has not been previously linked to AD. To date, none of these miRNAs have been specifically associated with CSF, suggesting that they may represent novel biomarkers for this fluid.

Taken together, these findings highlight the complex and fluid-specific behavior of miRNAs in AD, with some miRNAs showing consistent regulation across blood and CSF, while others display divergent patterns. These observations underscore the importance of comparative, multi-fluid approaches to better capture disease-related molecular alterations and to improve the identification and interpretation of reliable biomarkers.

### 4.3. EV-Derived miRNAs as Promising Biomarkers for AD Neuropathology

Extracellular vesicles (EVs), which include exosomes (30–150 nm) and microvesicles (100–1000 nm), are lipid bilayer-enclosed particles secreted by nearly all cell types. These EVs are widely distributed across biological fluids, including blood, CSF, urine, and saliva [104] and have emerged as key mediators of intercellular communication by transporting bioactive cargo (i.e., proteins, lipids, mRNAs, miRNAs, and long non-coding RNAs) between cells. In AD, neurons, glial cells, and other brain-resident cells release EVs that contain pathological proteins such as Aβ and tau. Increasing evidence indicates that some EV subsets can cross the BBB and appear in peripheral circulation, providing a potential window into CNS processes. Although it has been suggested that this transport could be mediated via receptor-mediated transcytosis, glymphatic clearance pathways, or increased BBB permeability under pathological conditions [105,106], the exact mechanisms governing it are the subject of ongoing investigation.

Our findings indicate that a substantial proportion of the detected miRNAs (65.9%) is likely associated with exosomal fractions. Notably, a total of 481 miRNAs were identified in EVs, of which 360 were exclusively reported in exosome-based studies, further supporting the notion that EVs represent a major reservoir of circulating miRNAs in AD (Supplementary Figure S3). This observation is of particular relevance as EV-derived miRNAs are thought to be more stable and reflective of CNS processes, given their ability to cross the BBB and protect their cargo from degradation. Nevertheless, additional studies will be required to better characterize the cellular origin and functional implications of these EV-associated miRNAs.

### 4.4. Potential Inflammatory/Apoptosis Disruption of Altered miRNAs

Most of the top potential targets identified herein directly regulate the neuronal survival/apoptosis pathway (BCL2, BCL2L11 (BIM), BAX, AKT1, PTEN, FOXO1). In this context, it is especially relevant to indicate that among the 941 unique proteins that may constitute targets of miRNAs common to both blood and CSF, BCL2 and BCL2L11 (BIM) are among the top potentially regulated proteins. Both proteins are involved in cell survival. Specifically, the anti-apoptotic protein BCL2 protects cells from death by binding to and inhibiting pro-apoptotic proteins like BCL2L11 [107]. This interaction prevents the activation of mitochondria-mediated apoptosis [108]. Additionally, BCL2L11 activates BAX, identified herein as a potential target, and BAK [109], which, as a result, induces the release of cytochrome c from mitochondria and promotes apoptosis [110,111,112]. Therefore, the proteins BCL2, BCL2L11, and BAX display a fine-tuned interrelationship that controls cell apoptosis. Potential disruption of any of these proteins, as our research strongly suggests, would end with an imbalance of cellular apoptosis. In the context of AD, strong evidence demonstrates that these proteins are altered or display a protective role [108,113]. Additionally, PTEN/FOXO1-mediated inhibition of PI3K-AKT signaling promotes apoptosis by enhancing the expression of pro-apoptotic effectors while simultaneously attenuating BCL2-dependent survival pathways, and NFKB1, STAT3, MAPK1, and SIRT1 modulate the apoptosis-survival balance amid defective autophagy-all converging in Aβ/tau-induced neuronal loss [114], thereby shifting the balance toward programmed cell death.

It is also known that BCL2 regulates autophagy through ATG5 [115,116] and BECN1 [107], also identified herein as potential targets. Autophagy has been described as mainly reduced in brains [117] and plasma [118] of AD patients, as well as in brain tissues of mouse models of aging [119], which is in concordance with the identification in the literature of a reduction in miR-30d-5p and miR-9-5p that potentially target ATG5 and BECN1 mRNAs.

Any dysregulation of the mentioned proteins as a potential consequence of miRNAs’ altered expression may lie behind the altered balance between neuronal survival, apoptosis, and autophagy, thereby contributing to neurodegeneration in AD and to a chronic inflammatory state of the brain. In this sense, it is also important to mention that a second group of altered miRNAs, mostly downregulated in blood and CSF of AD patients, constitutes part of the inflammatory/immune regulatory pathways (IL6, IFNG, NFKB1, MMP9, NOTCH1, NOTCH2). Increased levels of most of them have been observed in relation to AD, such as IL6 [120], NFKB1 [121], MMP9 [122], and NOTCH1 [123]. Therefore, reduction in the regulatory mechanism driven by miRNA may contribute somehow to the increased expression of these proteins.

The common miRNAs identified in both blood and CSF thus target key regulators of essential AD processes, including apoptosis and autophagy, positioning them as promising modulators of neurodegenerative progression and therapeutic targets.

### 4.5. Diseases and Proteins

When exploring the diseases associated with the miRNAs identified in our analysis, numerous cancers emerged prominently. This observation may not be unexpected, as many of these miRNAs are involved in fundamental molecular pathways that are shared between cancer and AD, including apoptosis, cell cycle regulation, autophagy, and inflammatory signaling. Thus, the frequent association with cancer likely reflects their pleiotropic roles in core cellular processes and highlights the importance of interpreting enrichment analyses within a broader biological context, particularly in complex disorders such as AD. Their involvement in key pathogenic mechanisms suggests that they may play a critical role in disease progression and represent valuable targets for further investigation.

The evidence included in this review primarily supports miRNAs as candidate diagnostic/discriminative biomarkers, since most studies compared AD patients with cognitively healthy controls. By contrast, their use in preclinical detection, disease monitoring, treatment response, or prognosis remains limited. miRNA-based biomarkers should therefore be considered complementary rather than alternative to established AD biomarkers. Whereas CSF Aβ and tau and emerging blood-based biomarkers such as plasma p-tau and NfL reflect core AD pathology and neurodegeneration, miRNAs may provide an additional regulatory and pathway-related molecular layer. Blood-based miRNAs are attractive for repeated and large-scale testing because sampling is minimally invasive, although circulating profiles may be affected by peripheral tissues, systemic conditions, and pre-analytical variability. CSF-derived miRNAs may more closely reflect CNS-related molecular changes but require a more invasive sampling procedure. Demonstrating clinically meaningful added value will require standardized assays, direct multimarker comparisons, independent cohorts, and longitudinal validation. This review has some limitations that should be considered when interpreting the findings. The literature search was conducted using PubMed, which provides broad coverage of biomedical research, although some relevant studies indexed exclusively in other databases may not have been captured. In addition, methodological heterogeneity across the included studies limited the feasibility of a conventional quantitative meta-analysis. Differences among blood-derived sample types, including serum, plasma, whole blood, extracellular vesicles, and blood cells, may also contribute to variability in miRNA profiles and influence the observed consistency across studies. Additional heterogeneity may arise from differences in sample collection and processing procedures and miRNA normalization/reference strategies, which were not consistently reported across studies and could therefore not be systematically evaluated. Consequently, a direction- and consistency-based synthesis was used, and the findings should be interpreted as patterns of net directional support across heterogeneous studies rather than pooled estimates of effect size. In addition, these patterns should be considered within the context of biomarker-discovery and multi-candidate profiling literature.

## 5. Conclusions

Due to variability across biofluids, study designs, and detection methods, miRNAs are not currently considered reliable diagnostic biomarkers. However, the coexistence of common and fluid-specific miRNA signatures reinforces the need for integrated, comparative studies across biofluids, which are critical for improving biomarker discovery and enhancing our understanding of AD pathophysiology. This systematic review quantitatively investigated AD differential miRNA expression in blood and CSF, identifying 33 miRNAs dysregulated in both fluids of AD patients, showing consistent opposing and concordant blood–CSF signatures across studies. Specifically,16 miRNAs showed concordant direction and 17 exhibited discordant direction. Of these, miR-451a, miR-16-5p, and miR-29b demonstrate replicability and biological relevance in AD pathways and miR-532-5p, miR-365a-3p, miR-140-3p, miR-140-5p, miR-146b-5p, miR-454-3p, miR-140-3p, miR-584-5p and miR-30d-5p emerge as novel candidates. This approach moves beyond descriptive biomarker lists, highlighting miRNAs with biological plausibility that hold significant potential as both diagnostic biomarkers and therapeutic targets. Given that AD continues to represent a major global health burden, particularly in the aging population, further research should prioritize harmonized, cross-cohort validation, biofluid-specific analysis, and longitudinal designs to confirm these miRNAs as robust AD biomarkers and to better elucidate their contribution to AD pathogenesis.

## Data Availability

No new data were created or analyzed in this study. Data sharing is not applicable to this article.

## Supplementary Materials

The following supporting information can be downloaded at: https://www.mdpi.com/article/10.3390/ijms27219506/s1.
